# Single Microdroplet Breakup-Assisted Viscosity Measurement

**DOI:** 10.3390/mi13040558

**Published:** 2022-03-31

**Authors:** Yeongseok Jang, Hwabok Wee, Jonghyun Oh, Jinmu Jung

**Affiliations:** 1 Department of Mechanical Design Engineering, Jeonbuk National University, Jeonju 54896, Korea; ysjang@jbnu.ac.kr; 2 Department of Orthopaedics & Rehabilitation, College of Medicine, Pennsylvania State University, Hershey, PA 17033, USA; huw16@psu.edu; 3 Department of Nano-Bio Mechanical System Engineering, Jeonbuk National University, Jeonju 54896, Korea

**Keywords:** microviscometer, shear-thinning liquid, microdroplet, biopolymer viscosity

## Abstract

Recently, with the development of biomedical fields, the viscosity of prepolymer fluids, such as hydrogels, has played an important role in determining the mechanical properties of the extracellular matrix (ECM) or being closely related to cell viability in ECM. The technology for measuring viscosity is also developing. Here, we describe a method that can measure the viscosity of a fluid with trace amounts of prepolymers based on a simple flow-focused microdroplet generator. We also propose an equation that could predict the viscosity of a fluid. The viscosity of the prepolymer was predicted by measuring and calculating various lengths of the disperse phase at the cross junction of two continuous-phase channels and one disperse-phase channel. Bioprepolymer alginates and gelatin methacryloyl (GelMA) were used to measure the viscosity at different concentrations in a microdroplet generator. The break-up length of the dispersed phase at the cross junction of the channel gradually increased with increasing flow rate and viscosity. Additional viscosity analysis was performed to validate the standard viscosity calculation formula depending on the measured length. The viscosity formula derived based on the length of the alginate prepolymer was applied to GelMA. At a continuous phase flow rate of 400 uL/h, the empirical formula of alginate showed an error within about 2%, which was shown to predict the viscosity very well in the viscometer. Results of this study are expected to be very useful for hydrogel tuning in biomedical and tissue regeneration fields by providing a technology that can measure the dynamic viscosity of various prepolymers in a microchannel with small amounts of sample.

## 1. Introduction

Prepolymers have often been referred to as fully polymerizable chemical intermediates. These prepolymers have been widely utilized for preferred property modification and enhancement of polymers [1,2,3,4,5,6]. As a prerequisite for their applications, it is necessary to know a variety of property parameters of prepolymer solutions. One of the important property parameters is viscosity. With recent progress in bioengineering and biomedical fields, precise, rapid, and direct viscosity measurements of small volumes (from micro- to nano-volume) for non-Newtonian fluids are of interest to researchers [7,8,9,10,11]. In particular, the viscosity of hydrogels, which are widely used in tissue engineering and regenerative medicine, is known as a very important variable for forming the extracellular matrix (ECM), as well as being closely related to cell viability in ECM [12,13,14,15,16,17,18]. To address these trendy issues, diverse microfluidic devices have been applied for viscosity measurements of small volumes of less and more viscous prepolymer solutions in relationship to frictional resistance between two adjacent microfluid layers [19,20,21,22,23]. Nevertheless, small-volume viscosity measurements of prepolymer solutions remain an experimental challenge. 

Viscosity measurements of viscous samples in microscale for clinical analysis purposes have steadily been studied using various working principles. Chevalier et al. (2008) presented a micromachined capillary-based on-chip rheometer for wall shear stress and shear rate measurements on silicon oil and ethanol-based nanofluids [24]. Morhell et al. (2013) developed a microviscometer for analyzing transient responses of fluids in a single-channel glass microfluidic chip for precise viscosity measurements [25]. Solomon and Vanapalli (2014) reported a multiplexed viscometer using the flow-comparator technique to measure the viscosity as a function of shear rate for several samples simultaneously [26]. Sankaran et al. (2016) introduced a 3D-printed optofluidic microviscometer for rapid and automated measurements of milk adulteration with a high accuracy of 0.95 [27]. Deshmukh et al. (2016) developed a novel high-throughput viscometer using transient flow of a complex fluid through pipettes [28]. Maezban et al. (2017) employed a 3D-suspended polymeric microfluidic system for detecting changes in dynamic viscosity and density during fluid processes [29].

In this paper, we introduced a microviscometer with the simplest flow-focusing method that could directly measure the viscosity of a prepolymer sample on a microscale using characteristics of necking fluid during the microdroplet generation process. In the dripping regime, both the squeezed shape and segment size are completely dependent on the viscosity of the prepolymer solution. Polydimethylsiloxane (PDMS)-based flow-focusing microdevice was fabricated using photolithography and soft lithography. Two prepolymer solutions of alginate and GelMA (gelatin methacrylate) were applied for viscosity measurements according to different concentrations. GelMA is a photopolymerizable biomaterial in which methacrylate is conjugated to an amine group in gelatin, and is widely used in tissue engineering applications due to its excellent cell compatibility. In addition, alginate, a natural polymer, has been used as biomaterial to complement the mechanical properties of GelMA. These two biomaterials are in the spotlight as materials for constructing the extracellular matrix in the fields of biomedical and bioprinting, but analysis of viscosity closely related to cell viability is required. Shear-thinning liquid shape and microdroplet controlled by the flow rate of mineral oil were observed under a microscope to characterize the viscosity of the thinning liquid. The break-up shape of the dispersed phase was measured and analyzed in terms of various lengths. By obtaining a base formula based on the measured length, a parameter that could predict the viscosity of the prepolymer was found and a method for applying it was presented.

## 2. Materials and Methods

### 2.1. Prepolymer Solution Preparation

In order to synthesize GelMA, dimethyl sulfoxide (Sigma Aldrich, St. Louis, MO, USA) was mixed with 5 g of gelatin (Sigma Aldrich, St. Louis, MO, USA). The mixture was heated up to 50 °C with continuous stirring. After 0.5 g of 4-(dimethlyamino)-pyridine (Sigma Aldrich, St. Louis, MO, USA) was dissolved with the solution, 2 mL of glycidyl methacrylate (Sigma Aldrich, St. Louis, MO, USA) was added to the solution at a constant flow rate of 0.5 mL/min with vigorous stirring. The reaction was kept for two days under a dry N_2_ gas environment. And the solution was filtered using a membrane (molecular weight 12,000 to 14,000) with deionized water at 40 °C for 1 week. The deionized water was replaced once a day. A lyophilization-induced aggregated porous solid was obtained and stored at −80 °C. GelMA prepolymer solutions at 3 wt%, 5 wt%, and 8 wt% were prepared. Sodium alginate (Sigma Aldrich, St. Louis, MO, USA) with an average molecular weight between 12,000 and 40,000 was dissolved in deionized water to prepare concentrations of 0.1 wt%, 0.3 wt%, 0.5 wt%, 0.7 wt%, and 1 wt%, respectively. Mineral oil (Sigma Aldrich, St. Louis, MO, USA) and 25 wt% of Span 80 (Sigma Aldrich, St. Louis, MO, USA) were purchased and mixed together to increase the viscosity of the mineral oil for emulsification.

### 2.2. Microfluidic Device Fabrication

The microfluidic microdroplet device fabrication method has been described in a previous paper [30]. In brief, SU-8 (Microchem Inc., Newton, MA, USA) as a negative photoresist was spin-coated onto a silicon wafer and then baked at 95 °C. Micropatterns for the microfluidic device were generated under UV exposure using a photomask. After baking at 100 °C, the wafer was developed at room temperature, rinsed with isopropanol three times, and dried using N_2_. The silicon master for duplication of the microfluidic device was prepared. The mixture of silicone elastomer base and curing agent (10:1) (Sylgard 184 Silicone Elastomer Kit, Dow Corning, Midland, MI, USA) was poured on the silicon master and degassed under vacuum. After curing at 80 °C for 2 h, the PDMS was detached. Holes for three inlets and one outlet were punched out. The PDMS was then permanently bonded with a glass slide under oxygen plasma (CUTE, Femtoscience, Kyounggi, South Korea) for 1 min to fabricate the microfluidic device.

### 2.3. Experimental Procedures

The prepolymer solution was injected into the center inlet of the microfluidic device using a syringe pump (PHD2000, Harvard Apparatus, Boston, MA, USA) via a Tygon tube. Mineral oil mixed with Span 80 was pumped to the other two inlets using a syringe pump. The prepolymer solution and mineral oil were loaded with two separate 1 mL syringes. The mineral oil was used as the continuous fluid, while the prepolymer solution acted as the thinning (dispersed) fluid due to shear force induced by the mineral oil. The flow rate of the continuous phase ($Q_{c}$) mixed with the mineral oil and Span 80 was fixed at 500 μL/h. Flow rates of the prepolymer ($Q_{d}$) were adjusted to be 200, 400, and 600 μL/h, respectively. As the shear force changed during the microdroplet generation, the thinning fluid showed different behaviors. The non-Newtonian behavior of the prepolymer solution was characterized by measuring the width of the thinning fluid necking zone. The viscosity of the prepolymer solution was calibrated with two variables. The microfluidic device was observed under an inverted microscope (CKX41, Olympus Co., Tokyo, Japan). Snap images extracted based on the acquired video were analyzed using ImageJ/Fiji software Ver. 1.53p (https://imagej.net/software/fiji/ (accessed on 28 March 2022)). All experiments were repeated three times for each concentration of the prepolymer. Image analysis was performed by randomly extracting 10 images when the droplet broke up. Results are presented as mean ± standard deviation (SD).

## 3. Results and Discussion

**Figure 1:**
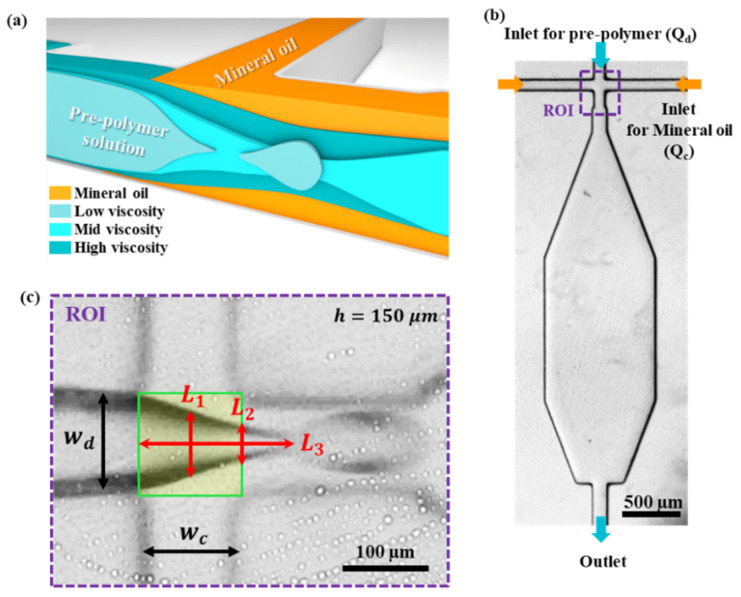
(**a**) Schematic of microdroplet breakup according to various viscosities of the disperse phase. (**b**) Microscopic image of a microviscometer (scale bar = 500 μm). (**c**) Working mechanism of microviscometer (scale bar = 100 μm).

Figure 1 shows the microdroplet generator’s viscosity measurement mechanism and device. The schematic in Figure 1a shows a cross section of the region where the continuous phase and the disperse phase intersect in the flow-focusing microdroplet generator channel. Mineral oil is run in the continuous phase with Span 80. The prepolymer solution is run in the disperse phase. When the prepolymer solution has low viscosity, a dripping regime occurs. When the prepolymer solution has high viscosity, a jetting regime occurs. Figure 1b shows an inverted microscope image of the microdroplet generator chip fabricated through soft lithography. It consists of two continuous-phase inlets, one disperse-phase inlet, and one outlet. Figure 1c shows an enlarged picture of the region of interest (ROI), which is the cross junction in Figure 1b. At the cross junction, the channel width of the continuous phase ($w_{c}$) and the channel width of the disperse phase ($w_{d}$) are each 100 μm. The height of the channel is 150 μm. Viscosity was calculated using measured lengths *L*_1_, *L*_2_, and *L*_3_ using a microdroplet generator. The length of *L*_1_ represents the vertical line of the disperse phase at the center of the cross junction (green box). The length of *L*_2_ represents the length of the vertical line at the point where the right edge of the cross junction (green box) and the disperse phase meet. The *L*_3_ length is defined as the length from the left starting point of the cross junction (green box) to the moment when the dripping regime occurs. 

**Figure 2:**
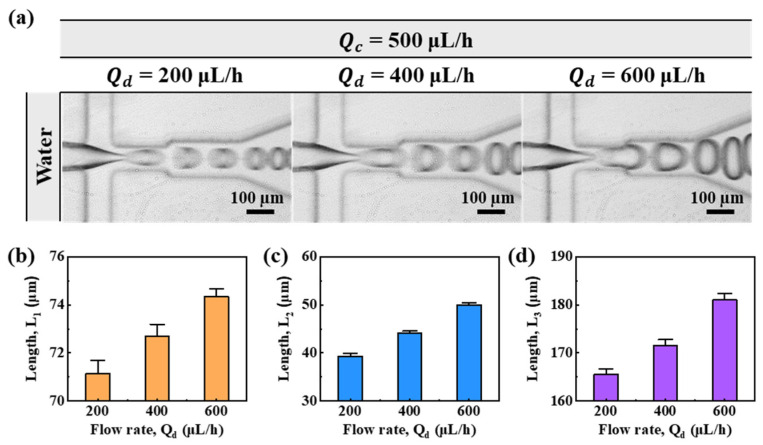
(**a**) Flow-focused microdroplet generation microscopic image according to the flow rate of water (*Q_d_*). (**b**–**d**) Lengths (*L*_1_, *L*_2_, and *L*_3_) at each location according to the flow rates of water (*Q_d_*).

Figure 2 shows microdroplet generating behavior and lengths of *L*_1_, *L*_2_, and *L*_3_ according to the flow rate of water when the flow rate of the continuous phase ($Q_{c}$) is 500 μL/h. Figure 2a shows microdroplet generating behavior according to the flow rate of water. When the flow rate of the disperse phase ($Q_{d}$) was 200 μL/h, the disperse phase at the cross junction showed the sharpest appearance. Disperse-phase breakup occurred immediately after the cross junction. The size of the generated microdroplet was the smallest and the generation rate was the fastest. When $Q_{d}$ was 600 μL/h, the disperse phase at the cross junction was thicker than that when $Q_{d}$ was 200 μL/h. The length of dripping was slightly longer and the size of the droplet was the largest. Figure 2b–d shows the lengths of *L*_1_, *L*_2_, and *L*_3_ according to the flow rate of water. In Figure 2b, the length of *L*_1_ was measured to be 71.15 ± 0.54 µm, 72.69 ± 0.51 µm, and 74.34 ± 0.33 µm at flow rates of 200, 400, and 600 μL/h, respectively. As the flow rate of the disperse phase increased, the length of *L*_1_ increased linearly. In Figure 2c, the length of *L*_2_ was measured to be 39.31 ± 0.66 µm, 44.08 ± 0.48 µm, and 50.00 ± 0.50 µm at flow rates of 200, 400, and 600 μL/h, respectively. The length trend of *L*_2_ was similar to that of *L*_1_. In Figure 2d, the length of *L*_3_ was measured to be 165.51 ± 1.16 µm, 171.58 ± 1.21 µm, and 181.03 ± 1.43 µm at flow rates of 200, 400, and 600 μL/h, respectively. In the case of water, all length measurements were within a 2% error range.

**Figure 3:**
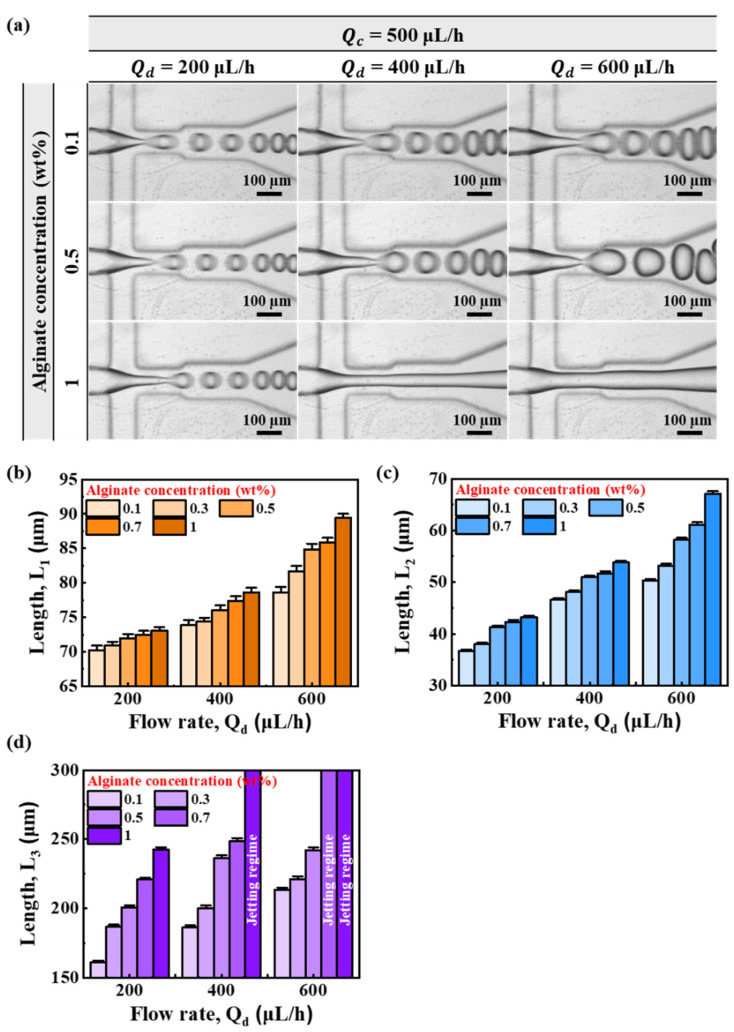
(**a**) Microscopic image of microdroplet generation by flow rate according to alginate concentration. (**b**–**d**) Lengths (*L*_1_, *L*_2_, and *L*_3_) according to flow rate by concentration of alginate.

Figure 3a shows flow behavior as a function of flow for alginate concentration. When $Q_{d}$ was 200 μL/h, as the concentration of alginate increased from 0.1 wt% to 1 wt%, there was a tendency to move from a dripping regime to a transition regime. When $Q_{d}$ was 400 μL/h, a jetting regime was seen when the concentration of alginate was 1 wt%. When $Q_{d}$ was 600 μL/h, the disperse-phase flow as a whole was unstable. When the alginate concentration was 1 wt%, it showed a thicker jetting regime at $Q_{d}$ = 600 μL/h than that at $Q_{d}$ = 400 μL/h. Figure 3b–d shows lengths of *L*_1_, *L*_2_, and *L*_3_ according to the flow rate for each concentration of alginate. Overall, lengths of *L*_1_, *L*_2_, and *L*_3_ tended to gradually increase as $Q_{d}$ increased. Lengths also increased slightly as the concentration of alginate in each flow group increased. In the low $Q_{d}$ section, the length change according to alginate concentration was not significant. However, in the high $Q_{d}$ section, the length according to the alginate concentration showed a big difference. In particular, as shown in Figure 3d, when $Q_{d}$ was 400 and 600 μL/h, respectively, the jetting regime was shown at 1 wt% of alginate concentration. For all $Q_{d}$ conditions, the error of the length was within the 2% range.

**Figure 4:**
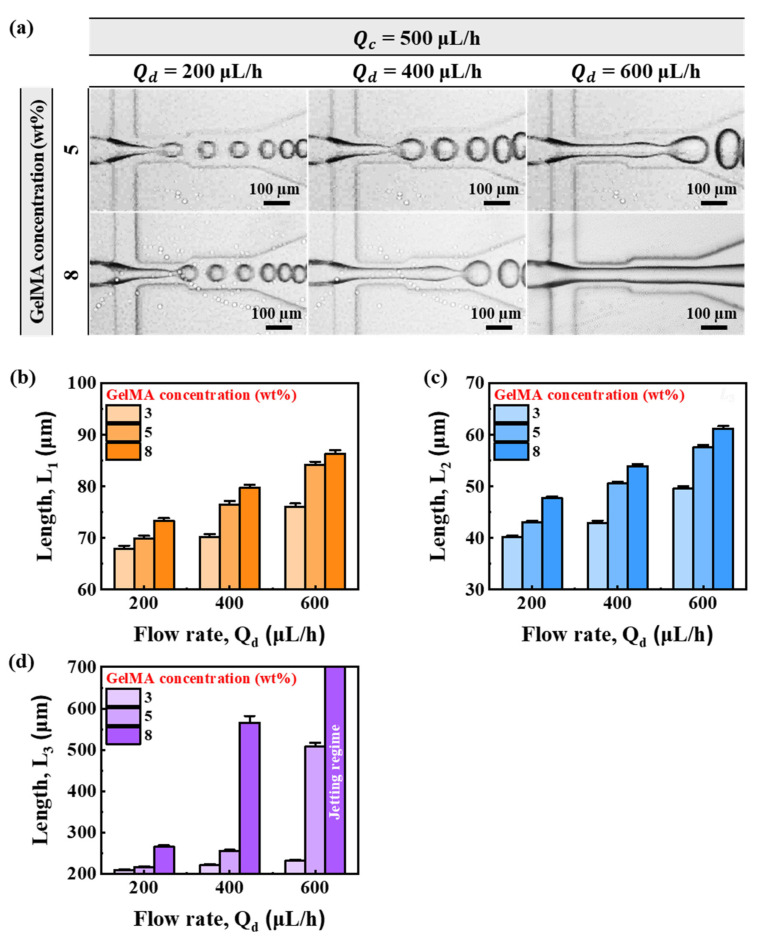
(**a**) Microscopic image of microdroplet generation by flow rate according to GelMA concentration. (**b**–**d**) Lengths (*L*_1_, *L*_2_, and *L*_3_) according to flow rate by concentration of GelMA.

Figure 4a shows flow behavior as a function of flow for GelMA concentrations of 5 wt% and 8 wt%. Overall, as $Q_{d}$ was higher, the behavior changed from dripping regime to jetting regime. In addition, the higher the concentration of GelMA, the more unstable the disperse-phase flow. In particular, GelMA concentration of 8 wt% under $Q_{d}$ = 400 μL/h and 5 wt% of GelMA at $Q_{d}$ = 600 μL/h had very long transition lengths. At $Q_{d}$ = 600 μL/h with GelMA concentration of 8 wt%, a jetting regime was observed. Figure 4b–d shows lengths of *L*_1_, *L*_2_, and *L*_3_ according to flow rate for each concentration of GelMA. As with the alginate prepolymer, the lengths of *L*_1_, *L*_2_, and *L*_3_ gradually increased with higher $Q_{d}$. The length also increased with higher concentrations of GelMA in each flow group. However, in the case of *L*_3_ length, when $Q_{d}$ was 400 μL/h, the length of the GelMA prepolymer at 8 wt% increased sharply. In the end, when $Q_{d}$ was 600 μL/h, 8 wt% of the GelMA prepolymer showed a jetting regime form.

The microdroplet formation process can be described as the capillary number ($Ca_{c}$) = ($\frac{\mu_{c}V_{c}}{\sigma })$ of the continuous-phase fluid, where $\mu_{c}$ is the dynamic viscosity of the continuous phase, $V_{c}$ is the fluid velocity of the continuous phase, and σ is the surface tension. The moving continuous phase exerts a drag force on the dispersed phase, causing a transition to jetting at a certain threshold of continuous-phase velocity [31,32]. In our experiment, the flow rate of the continuous-phase fluid was applied as an independent variable. However, under the same viscosity conditions of the dispersed phase, as the flow rate of the dispersed phase increased, the velocity of the continuous phase increased in the limited space of the cross junction. In other words, as $V_{c}$ increased, $Ca_{c}$ exceeded a certain threshold, resulting in a jetting regime. Therefore, an increase in $Ca_{c}$ caused increases of the measured lengths *L*_1_, *L*_2_, and *L*_3_ of the dispersed phase.

We verified the dynamic viscosity of the prepared prepolymer through a rheology analyzer to predict the viscosity based on the measured *L*_1_, *L*_2_, and *L*_3_ lengths. Results are shown in Table 1.

**Figure 5:**
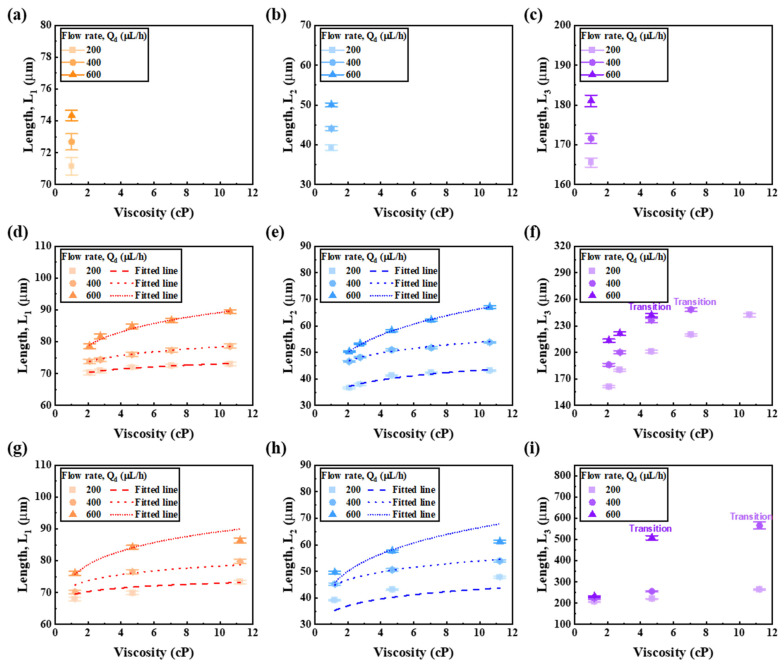
(**a**–**c**) Length with viscosity for flow rate of water. (**d**–**f**) Length and derived fitting line with viscosity for flow rate of alginate. (**g**–**i**) GelMA viscosity curve for applied fitting line and length.

Figure 5 shows the redrawing of the measured *L*_1_, *L*_2_, and *L*_3_ based on the dynamic viscosity analyzed in Table 1. Figure 5a–c shows each of *L*_1_, *L*_2_, and *L*_3_ according to the viscosity of water. It can be seen that for water with single viscosity, lengths of *L*_1_, *L*_2_, and *L*_3_ are affected by the flow rate of the disperse phase. Figure 5d–f shows a fitted line as well as *L*_1_, *L*_2_, and *L*_3_ as a function of alginate viscosity versus flow rate. Overall, the trend for length with viscosity was logarithmic. Excluding *L*_3_, where the jetting regime occurred, an expression that could predict the viscosity along the length from the alginate curve was derived. The empirical formulas derived from the data in Figure 5d,e are the same as Equations (1) and (2), respectively.

(1) $$L_{1,m}=L^{*}\mu_{s}{}^{\epsilon }$$

(2) $$L_{2,m}=L^{*}\mu_{s}{}^{\epsilon }$$

Equations (1) and (2) expressed again for dynamic viscosity of sample ($\mu_{s}$) as follows:

(3) $$\mu_{s}={\left(\frac{L_{1,m}}{L^{*}}\right)}^{\frac{1}{\epsilon }}$$

(4) $$\mu_{s}={\left(\frac{L_{2,m}}{L^{*}}\right)}^{\frac{1}{\epsilon }}$$

where $L_{1,m}$ and $L_{2,m}$ represent the measured lengths of *L*_1_ and *L*_2_, respectively. L* and ϵ represent respective constants for the fitting curve. Table 2 shows parameter values for these equations. Equation (1) shows very good fitting with R-squared values (R^2^) of 0.973, 0.998, and 0.990 at flow rates of 200, 400, and 600 μL/h, respectively, shown in Figure 5d. Equation (2) in Figure 5e showed very small errors as R^2^ (0.940, 0.975, and 0.999 at flow rates of 200, 400, and 600 μL/h, respectively). 

We substituted into GelMA using viscosity Equations (3) and (4) along the length from the alginate prepolymer. Figure 5g–i shows *L*_1_, *L*_2_, and *L*_3_ as a function of GelMA viscosity versus flow rate. Equation for Equation (3) is substituted in Figure 5g and Equation (4) is substituted in Figure 5h. Figure 5f,i could not derive a fitting line due to the jetting regime. In Figure 5g,h, the length error increased in the high viscosity range. However, for $Q_{d}$ = 400 μL/h, Equations (3) and (4) predicted the viscosity versus length almost accurately.

**Figure 6:**
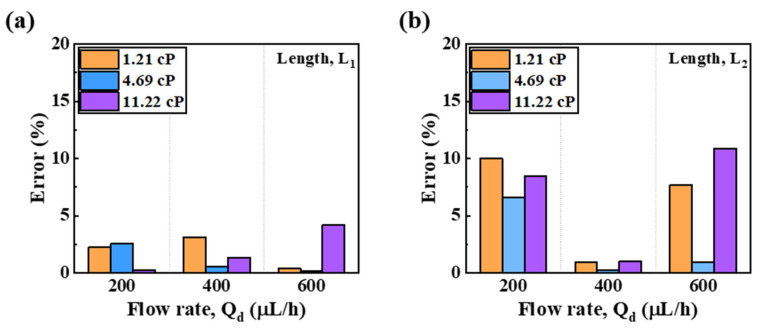
Errors between the measured and calculated lengths for GelMA (**a**) *L*_1_ length and (**b**) *L*_2_ length.

Figure 6a shows the error for the fitting line of Equation (3) from the measured *L*_1_ length of the GelMA prepolymer. For all flow rates at each viscosity of GelMA, the curve of Equation (3) had an error range from 0.14% to 4.18%. Figure 6b shows the error for the fitting line of Equation (4) from the measured *L*_2_ length of the GelMA prepolymer. Based on the fitting line of Equation (4), the length error of *L*_2_ had an error range from 0.23% to 10.88% for all flow rates. In particular, when $Q_{d}$ was 200 μL/h, both viscosity models had large error values for Equation (4). When $Q_{d}$ was 600 μL/h, the high-viscosity model showed a larger error value. The reason why the *L_2_* length error was larger than the *L*_1_ length error of the GelMA solution in a specific viscosity range was because unstable dripping and jetting regime of the dispersed phase occurred at the cross junction of the microdroplet generator chip. In addition, the error rate was large because the space constraint of the cross junction according to the viscosity of the dispersed phase made $Ca_{c}$ unstable. However, overall, for *L*_1_ and *L*_2_ lengths, Equations (3) and (4) represented the best predictors of viscosity for GelMA when $Q_{d}$ was 400 μL/h.

## 4. Conclusions

We described a new microviscometer that could directly measure the viscosity of a small amount of polymer sample by analyzing the necking phenomenon of the disperse-phase fluid generated at the cross junction from a length perspective using a flow-focusing microdroplet generator chip. The PDMS-based flow-focusing microdroplet generator chip was fabricated by photolithography and soft lithography. Alginate and GelMA were used to measure the viscosity at different concentrations under the same flow rate of the continuous phase. Lengths were carefully measured from various viewpoints. Alginate viscosity and GelMA viscosity were analyzed beforehand. The viscosity formula derived based on the length of the alginate prepolymer was applied to GelMA. It was found that the viscosity formula derived with alginate for some flow intervals ($Q_{d}$ = 400 μL/h) could perform predictions very well with 2% error. In addition, our study proposed a method that could measure a wide viscosity range. Moreover, it is more accurate than current methods. Results of the present study are expected to be very useful for hydrogel tuning in biomedical and tissue regeneration fields by providing a technology that could measure the dynamic viscosity of various prepolymers in a microchannel with small amounts of sample.

## Figures and Tables

**Table 1:** Dynamic viscosity according to the concentration of each sample.

Sample	Concentration (wt%)	Viscosity, μ (cP)
Water	0	1.002
Alginate	0.1	2.079
	0.3	2.751
	0.5	4.659
	0.7	7.056
	1	10.609
GelMA	3	1.210
	5	4.690
	8	11.220

**Table 2:** Parameters for derived Equations (1) and (2).

Parameters	Length, *L*_1_			Length, *L*_2_		
	*Q_c_* = 500 μL/h			*Q_c_* = 500 μL/h		
	*Q_d_* = 200 μL/h	*Q_d_* = 400 μL/h	*Q_d_* = 600 μL/h	*Q_d_* = 200 μL/h	*Q_d_* = 400 μL/h	*Q_d_* = 600 μL/h
$L^{*}$	69.190	71.784	74.669	34.600	44.036	44.253
ϵ	0.023	0.038	0.077	0.097	0.088	0.177
R^2^	0.973	0.998	0.990	0.940	0.975	0.999

## Data Availability

Not applicable.
